# P-1710. Prescribing Patterns for *Clostridioides difficile* Treatment in Outpatient Setting Using the Collaboration to Harmonize Antimicrobial Registry Measures (CHARM)

**DOI:** 10.1093/ofid/ofae631.1876

**Published:** 2025-01-29

**Authors:** Tiasha Nandi, Kathryn M Pawlowski, Benjamin Pontefract, Michael Klepser, Minji Sohn

**Affiliations:** Ferris State University, College of Pharmacy, Big Rapids, Michigan; Ferris State University, College of Pharmacy, Big Rapids, Michigan; Ferris State University, Grand Rapids, Michigan; Ferris State University, Grand Rapids, Michigan; Ferris State University, Grand Rapids, Michigan

## Abstract

**Background:**

Clostridioides difficile infection (CDI) is a form of infectious diarrhea associated with antibiotic use. In 2021, the IDSA released guidelines for treatment of CDI, which recommended fidaxomicin for 10 days as the first line treatment and vancomycin as an acceptable alternate. It is unclear to what extent prescribers have adopted this recommendation. This study aimed to examine patients’ antibiotic exposure before developing CDI and describe the prescribing patterns for CDI treatment in outpatient settings.

**Table 1:**
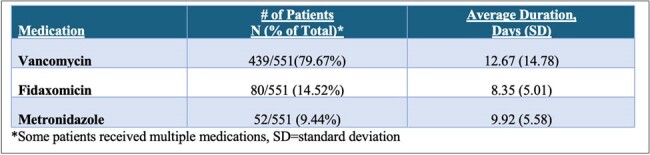
Frequency and Duration of Initial Clostridioides difficile Infection (CDI) Therapy

**Methods:**

This observational study used the CHARM database, which contains outpatient encounter data extracted from the medical records of nine health systems. Patients with CDI were identified using ICD-10 codes and medications commonly used for CDI. The data period was between July 2019 and June 2023. The frequency and duration of antibiotics used for CDI treatment and the percentage of patients receiving subsequent therapy were estimated. Antibiotics prescribed within 180 days prior to a diagnosis of CDI visit were captured.

**Figure 1:**
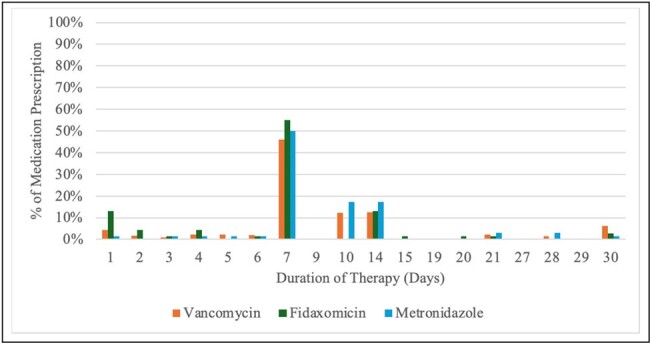
Duration Distribution of Initial Clostridioides difficile Infection (CDI) Therapy

**Results:**

A total of 551 patients with CDI were identified. Of those, 439 (80%) received vancomycin, 80 (15%) received fidaxomicin, and 52 (9%) received metronidazole. The average duration of therapy in days for vancomycin, fidaxomicin, and metronidazole was 12, 8, and 10, respectively (Table 1). The most common duration of therapy was 7-days for vancomycin (46%), fidaxomicin (41%), and metronidazole (50%) (Figure 1). After the initial treatment, 4% received a subsequent treatment within 3 weeks. Fluoroquinolone prescriptions had the highest prevalence of CDI (3.6 cases per 10,000 prescriptions). Of patients with an identified antibiotic 30 days prior to CDI, 38% had multiple courses of antibiotics.

**Conclusion:**

Receiving multiple courses of antibiotics is associated with developing CDI. The majority of patients with CDI received vancomycin treatment (the alternative recommended therapy). The average duration of therapy was longer than recommended. Metronidazole use is still common.

**Disclosures:**

**Minji Sohn, PhD**, Emergent BioSolutions, Inc.: Grant/Research Support

